# Allergic Non-Asthmatic Adults Have Regional Pulmonary Responses to Segmental Allergen Challenge

**DOI:** 10.1371/journal.pone.0143976

**Published:** 2015-12-07

**Authors:** Vanessa J. Kelly, Tilo Winkler, Jose G. Venegas, Mamary Kone, Daniel L. Hamilos, Roshi Afshar, Josalyn L. Cho, Andrew D. Luster, Benjamin D. Medoff, R. Scott Harris

**Affiliations:** 1 Divisions of Pulmonary and Critical Care and Rheumatology, Allergy and Immunology, Department of Medicine, Massachusetts General Hospital and Harvard Medical School, Boston, Massachusetts, United States of America; 2 Department of Anaesthesia, Critical Care and Pain Medicine, Massachusetts General Hospital and Harvard Medical School, Boston, Massachusetts, United States of America; 3 Center for Immunology and Inflammatory Diseases, Massachusetts General Hospital and Harvard Medical School, Boston, Massachusetts, United States of America; Telethon Institute for Child Health Research, AUSTRALIA

## Abstract

**Background:**

Allergic non-asthmatic (ANA) adults experience upper airway symptoms of allergic disease such as rhinorrhea, congestion and sneezing without symptoms of asthma. The aim of this study was to utilize PET-CT functional imaging to determine whether allergen challenge elicits a pulmonary response in ANA subjects or whether their allergic disease is truly isolated to the upper airways.

**Methods:**

In 6 ANA subjects, bronchoalveolar lavages (BAL) were performed at baseline and 24h after instillation of an allergen and a diluent in separate lung lobes. After instillation (10h), functional imaging was performed to quantify and compare regional perfusion, ventilation, fractional gas content (Fgas), and glucose uptake rate (Ki) between the baseline, diluent and allergen lobes. BAL cell counts were also compared.

**Results:**

In ANA subjects, compared to the baseline and diluent lobes, perfusion and ventilation were significantly lower in the allergen lobe (median [inter-quartile range], baseline vs. diluent vs. allergen: Mean-normalized perfusion; 0.87 [0.85–0.97] vs. 0.90 [0.86–0.98] vs. 0.59 [0.55–0.67]; p<0.05. Mean-normalized ventilation 0.89 [0.88–0.98] vs. 0.95 [0.89–1.02] vs. 0.63 [0.52–0.67], p<0.05). In contrast, no significant differences were found in Fgas between baseline, diluent and allergen lobes or in Ki. Total cell counts, eosinophil and neutrophil cell counts (cells/ml BAL) were significantly greater in the allergen lobe compared to the baseline lobe (all P<0.05).

**Conclusions:**

Despite having no clinical symptoms of a lower airway allergic response (cough and wheeze) allergic non-asthmatic subjects have a pulmonary response to allergen exposure which manifests as reduced ventilation and perfusion.

## Introduction

Allergen exposure in allergic non-asthmatic (ANA) adults causes upper airway symptoms such as sneezing, rhinorrhea and nasal and/or sinus congestion. In contrast, allergen exposure in allergic asthmatic (AA) subjects causes lower airway narrowing and pulmonary symptoms of wheeze and cough which may occur in concert with upper airway symptoms [1]. Despite differences in symptoms, both AA and ANA subjects have been shown to have similar lower airway cellular inflammatory responses to allergen challenge [2,3]. It remains unclear why similar cellular inflammatory responses invoke disparate clinical symptoms in AA and ANA subjects, and if allergen challenge in ANA subjects leads to alterations in regional lung function as has been shown during broncoconstriction in asthma [4,5]. One possibility is that allergen exposure in ANA subjects does not impact regional lung function.

Using functional PET-CT imaging in AA subjects, we have previously shown that segmental allergen challenge causes an allergic inflammatory cellular response in addition to regional physiological changes in the allergen exposed lobe compared to the control lobes [6]. Specifically, regional reductions in perfusion, ventilation, lung aeration and elevated glucose metabolism occurred in the allergen exposed lobe compared to the control lobes [6]. These findings suggest that the allergic inflammatory response in the lower airways of AA subjects results in clearly identifiable physiological changes that may account for the pulmonary symptoms in these subjects. Given that ANA subjects, following allergen exposure, exhibit symptoms of an upper airway allergic response without pulmonary symptoms, it is reasonable that they may not experience the reductions in perfusion, ventilation, lung aeration and elevated glucose metabolism that occur in AA subjects. However, whether the lower airway cellular inflammatory response in ANA subjects following allergen challenge manifests as alterations in regional physiologic function such as ventilation and perfusion remains unknown.

Our aim was to use PET-CT functional imaging (^13^NN-saline and ^18^F-Fluorodeoxyglucose, ^18^F-FDG) to determine whether, in ANA subjects, segmental allergen challenge causes regional reductions in perfusion, ventilation, lung aeration and elevated glucose uptake. We hypothesized that ANA subjects would elicit a cellular inflammatory response to segmental challenge but have no regional physiologic responses.

## Materials and Methods

### Study Protocol

The Institutional Review Board of the Massachusetts General Hospital approved the study and all six subjects (4 female) provided written informed consent (Protocol No. 2008-P000408). Subjects were not taking any medications other than loratadine for control of allergy symptoms, which was withheld for 12 h prior to the first bronchoscopy. The study consisted of a screening visit, two hospital visits and a bronchoscopy-imaging visit.

### Study Inclusion Criteria

Study inclusion criteria required a clinical history of allergic symptoms to cat or house dust mite with demonstrated positive skin prick test reactivity, age 18–50 years, no history of asthma and a documented lack of airways hyperresponsiveness (methacholine PC20 > 16 mg/ml). The PC20 describes the concentration of methacholine required to provoke a 20% decrease in FEV_1_ [7]. Caution was taken to exclude subjects who were asthmatic, including those who did not present with asthmatic symptoms. The initial phone screen investigated subjects’ respiratory history through a series of questions pertaining to past breathing difficulties. During the screening visit, subjects needed to demonstrate a normal baseline spirometry (FEV_1_ and FVC both >90% predicted). During the second visit, subjects needed to demonstrate a negative methacholine bronchial challenge test (PC20 > 16 mg/ml.) Subjects with allergic rhinitis were studied outside of symptomatic pollen seasons. Exclusion criteria also included subjects with a BMI > 35 kg/m^2^, pregnant (confirmed via a positive serum pregnancy test) or nursing women, subjects with previous radiation exposure in research studies within the previous 12 months, and subjects with any concomitant lung disease or co-morbidities such as diabetes or known cardiac disease. Subjects had to be non-smokers for the previous 5 years with a < 10 pack year smoking history, have no evidence of respiratory tract infection in the previous 6 weeks or antibiotic use for respiratory complaints within the month prior to the first bronchoscopy visit, no history of exposure to asbestos or silica, and no anti-histamine use in the 7 days prior to the screening. Subjects who were unable to lay flat for more than 2 hours, had previous immunotherapy with cat or house dust mite extract, and subjects with known allergy or hypersensitivity to ^18^F-FDG were also excluded.

### Screening Visit

The screening visit included a full medical history, baseline spirometry (Morgan Heated FVL, Haverhill, MA USA) and serum pregnancy testing. Allergen skin testing was performed to first establish atopy to cat dander or house dust mite and was followed by quantitative allergen skin prick testing using serial 3-fold dilutions of either cat dander (Cat) or house dust mite Dermatophagoides Pterynissinus (DP) allergen to establish the minimum allergen concentration required to elicit an inflammatory response (3 mm wheal diameter) [8], which was the concentration subsequently instilled during the segmental lobe challenge. All allergens were purchased from Greer Laboratories (Lenoir, NC USA).

### Bronchoscopy-Imaging Visit

The bronchoscopy-imaging visit included two bronchoscopies separated by 24 h and PET-CT imaging with ^18^F-FDG and ^13^NN-saline approximately ~10 h after the first bronchoscopy. The timing of the bronchoscopies, 24 hours apart, was specifically selected to capture the peak in the T-cell response [9,10]. It was not possible to perform the bronchoscopes and imaging at the same time due to constraints in the scheduling of bronchoscopies and PET-CT imaging. As a result, the closest possible time the imaging could be completed with respect to the bronchoscopies was 10 hours after the first bronchoscopy, which corresponds to ~12 hours prior to the second bronchoscopy.

**Fig 1:**
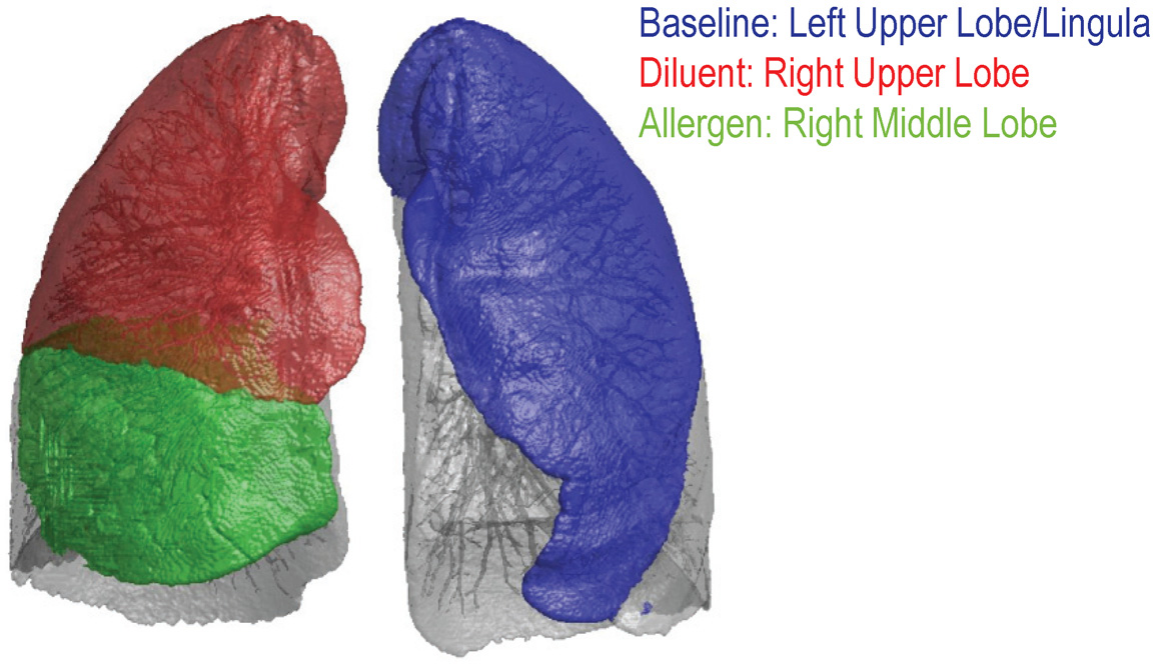
Lung image demonstrating the location of the different lung lobes. The left upper lobe (LUL) was used as the baseline (blue), the right upper lobe (RUL) was used as the diluent lobe (red) and the right middle lobe (RML) was the allergen exposed lobe (green).

The segmental allergen bronchoscopy methods were based on those developed by Lilly et. al. [8]. Each fiberoptic bronchoscopy was performed under conscious sedation with topical anesthesia (< 300 mg lidocane, total dose). During the first bronchoscopy a bronchial-alveolar lavage (BAL) was performed in the lingula/left upper lobe (LUL, Fig 1) with 120 mL of saline. The allergen (Cat or DP) was instilled (2 mL) in either the medial or lateral segment of the Right Middle Lobe (RML, Fig 1) at a concentration determined from quantitative skin prick testing. Also, due to the presence of a small amount of endotoxin within the instilled allergen solutions, 2ml of the respective diluent, which also contains the endotoxin (0.9% sodium chloride, 0.03% albumin and 0.4% phenol), was instilled in the anterior segment of the right upper lobe (RUL, Fig 1). The second bronchoscopy included a BAL performed on both the RUL and RML, each of 120 ml. The amount of endotoxin within the instilled cat allergen extract ranged from 5.0–6.5 endotoxin units/ml and for the DP allergen the range was 1.5–81 endotoxin units/ml. Endotoxin was measured in the undiluted commercial allergen extracts by kinetic turbidimetric chromogenic testing (Charles River Laboratories, Inc. Charleston SC). Please note, following the conclusion of this study, our group has since altered our segmental allergen technique to further reduce the risk of systemic allergic reaction. Specifically, the instilled DP allergen dose was reduced to 1/3 of the skin prick test threshold concentration as a result of hives developing in a subject in a parallel study.

### Image Instrumentation, Acquisition and Reconstruction

Prior to any imaging, subjects were instrumented with impedance plethysmography bands to monitor thoracic gas volume (SomnoStar PT, SensorMedics, Loma Linda, CA) and a peripheral intravenous catheter was inserted to allow for serum pregnancy test, delivery of the radioactive tracers and blood sampling during ^18^F-FDG imaging.

All images were obtained on a Biograph 64 PET-CT scanner (Siemans, Malvern, PA USA). Following instrumentation, a High-Resolution Computed Tomograph (HRCT) of the thorax was obtained (Xray tube current 160 mA, 120kVp) at Mean Lung Volume (MLV). MLV was defined as the average lung volume during relaxed tidal breathing in the supine position and was determined via impedance plethysmography for the 30 sec of breathing just prior to image acquisition. The HRCT scan was reconstructed using a B31 kernel yielding an in-plane image resolution of 0.9766×0.9766 mm, in-plane image size of 512×512 voxels, slice thickness of 0.75 mm and a beam pitch of 0.5 mm. The HRCT image was used for quantification of lung aeration, for the reconstruction of both the ^13^NN and ^18^F-FDG PET scans to correct for attenuation due to body tissues and for image segmentation (Apollo Workstation, Vida Diagnostics, Coralville, IA USA). Image segmentation yielded mask images of the lung and lobes.

A ^13^NN-Saline emission scan was performed following the HRCT scan. Specifically, at the onset of a 20–30 sec breath-hold at MLV, a bolus of ^13^NN-Saline was injected intravenously (11.2 [9.5–11.7] mCi). At the conclusion of the breath-hold, the subject was instructed to resume breathing for 3 min 10 s at the same frequency and tidal volume as recorded prior to the apnea (visual feedback provided via video goggles) while imaging of the ^13^NN washout for 3 min 10 s (total image time 3 min 40 s). The ^13^NN-saline emission scan allows for the assessment of regional pulmonary perfusion (Q) and specific ventilation (sV). The theoretical basis of this emission scan is the low solubility of nitrogen in tissues (partition coefficient λwater/air, 0.0015 at 37◦C), whereby on arrival of the ^13^NN-saline bolus into the pulmonary capillaries virtually all the tracer diffuses into the alveolar space on first-pass. Therefore the accumulation of ^13^NN in the alveolar space during the breath-hold period can be used as a measure of relative Q. With the resumption of breathing, the tracer is removed via ventilation and therefore the rate of removal of ^13^NN can be used to estimate regional sV.

^13^NN-saline PET scans were reconstructed using filtered back projection yielding dynamic scans with a voxel size of 5.3456×5.3456×2.025 mm, 128×128×81 voxels per frame and 16 frames (frame duration: 8×5 s, 3×10 s, 5×30 s). PET scans were filtered using a 3 dimensional moving average filter resulting in an effective image resolution of 10.125 mm.

Gas fraction (Fgas) was computed for each voxel by converting the HRCT density, Hounsfield units (HU) to Fgas, where Fgas = (density_blood_−density_voxel_)/ (density_blood_−density_air_), using density_blood_ = 65 and density_air_ = −1000.

The ^18^F-FDG PET scans were commenced following the intravenous infusion of ^18^F-FDG (10.1 [9.6–10.3] mCi). From the onset of the infusion, each dynamic PET scan was acquired for a total of 52 min 45 s duration. In order to calibrate the FDG image, venous blood samples were acquired during the FDG image at set time points, 5 min 30 s, 9 min 30 s, 25 min, 37 min and 42 min 30 s, as described previously [11]. The blood samples (~1 ml) were centrifuged and the activity in the isolated plasma assessed with a gamma counter. FDG imaging is based on FDG uptake by cells via the same mechanism as glucose but subsequent trapping within the cell as ^18^F-FDG-6-phosphate following phosphorylation by hexokinase results in accumulation within the cell in proportion to the cells’ metabolic rate allowing for the determination of the ^18^F-FDG net uptake rate (see Image Analysis below). ^18^F-FDG scans were reconstructed using filtered back projection with the same geometry as the ^13^NN-saline scans but with 36 frames (frame duration: 9×10 s, 3×15 s, 1×30s, 7×60s, 14×120, 1×300 s, 1×600 s).

### Image analysis

For all image analysis, the exposed lung lobe, instead of the exposed lung segment, was used to assess the influence of the allergen and the diluent and to establish a baseline condition. The use of lung lobes significantly reduces the potential error in lung segmentation.

Ventilation and Perfusion: Voxel by voxel specific ventilation (sV) and perfusion (Q) were computed from dynamic PET scans of ^13^NN-Saline kinetics as previously described [12] and were then used to determine the regional mean normalized sV and Q in the baseline, diluent and allergen lobes.

Lung Aeration: Using the HRCT, the mean regional lung aeration was computed in the baseline, diluent and allergen lobes as the average gas fraction (Fgas; gas volume/total volume) relative the mean Fgas of the whole lung.

Cellular Metabolic Rate: Cellular metabolic activity in the baseline, diluent and allergen lobes were measured as the net ^18^F-FDG uptake rate (Ki), calculated via the Patlak method [13,14]. Specifically, both the lung activity and the integral of the plasma activity were normalized to the plasma activity and then plotted against each other. When the transfer of tracer between the plasma and the intercellular compartments reached a plateau, the plot of normalized lung activity vs. the integral of plasma activity linearizes (~ 8–10 min after ^18^F-FDG administration). The slope of this linear portion of the curve was estimated using linear regression and reflects the ^18^F-FDG uptake rate (Ki).

### Cellular analysis

Cellular analysis was completed on each BAL sample as previously described [6]. Total cell counts per ml of collected BAL were computed along with differential cell counts per ml of collected BAL for eosinophils, neutrophils and mononuclear leukocytes.

### Statistical Analysis

Non-parametric statistics were used and reported as median [interquartile range], unless otherwise stated. Specifically, inter-subject differences in the allergen, diluent and baseline means of Q, sV, Fgas and cell counts were completed with analysis of variance on Ranks and Tukey pairwise tests (allergen vs. diluent, allergen vs. baseline, diluent vs. baseline). Statistical analysis was completed using SigmaPlot (Systat Software, San Jose, CA USA) and Matlab (Mathworks, Natick MA, USA). Statistical significance was accepted at P<0.05. No corrections were made for multiple comparisons.

## Results

Subject characteristics, allergen dosing and lung function, including percentage predicted values [15] are included in Table 1. All subjects had normal lung function with no evidence of airflow obstruction. To enable individual subjects to be tracked throughout the figures, each subject is identified by a unique marker, subject 1 circle, subject 2 square, subject 3 diamond, subject 4 triangle, subject 5 upside down triangle, subject 6 hexagon.

**Table 1:** Subject demographics, lung function and allergen dosing.

Subject	Gender	Age, yrs	Height, cm	BMI, kg/m^2^	FEV_1_, L (% pred.)	FVC, L (%pred.)	FEV_1_/FVC	PC20, mg/mL	Allergen	Dose ^#^
1	F	20	160.0	22.1	4.03 (126)	4.37 (123)	0.92	19.96	DP	370
2	M	28	185.4	25.1	4.76 (98)	5.76 (99)	0.83	20.64	DP	1111
3	F	33	165.1	28.5	3.64 (120)	4.51 (126)	0.81	>25	DP	41
4	M	49	180.3	34.7	3.42 (85)	4.31 (87)	0.79	>25	DP	370
5	F	24	177.8	21.5	3.65 (101)	4.49 (104)	0.81	>25	Cat	370
6	F	31	162.6	24.3	3.85 (128)	5.10 (145)	0.75	>25	DP	123
Median [IQR]	M:F 2:4	30 [25–33]	171.5 [163.2–179.7]	24.7 [22.7–27.6]	3.75 [3.64–3.99] (111 [99–125])	4.50 [4.40–4.95] (114 [100–125])	0.81 [0.80–0.82]	-	DP:Cat 5:1	370 [185–370]

^#^ Dose units are Bioequivalent Allergy Units (BAU)/ml for cat dander and Allergy Units (AU)/ml for the DP extract. Two mL of extract was instilled during each segmental allergen challenge.

### PET-CT functional outcomes

**Fig 2:**
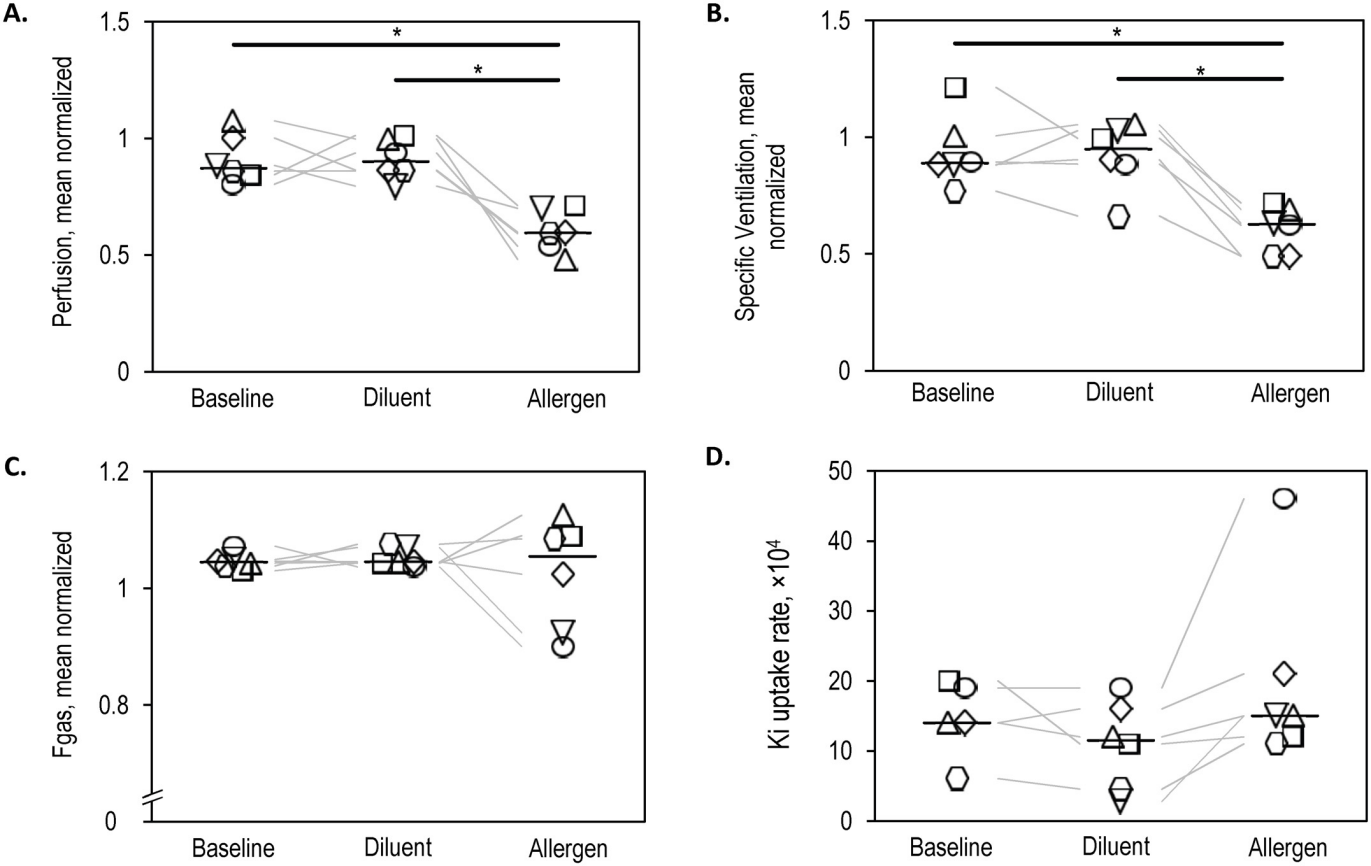
Physiological changes in response to segmental allergen challenge. Both relative perfusion (A) and specific ventilation (B) were significantly lower in the allergen lobe compared to the baseline and diluent lobes. (C) However, overall the fractional gas content was not different between the baseline, diluent and allergen segments. (D) Ki uptake rate was also not different between the baseline, diluent and allergen segments.

Both the relative Q and sV were significantly lower in the allergen lobe compared to the diluent lobe (P<0.05) and baseline lobes (P<0.05 Fig 2). No differences in Fgas were found between the baseline, diluent or allergen lobes (Fig 2). For one subject the baseline lobe ^18^F-FDG analysis was unable to be completed due to a local edge effect as a result of patient movement and was therefore excluded from the statistical analysis. Comparison of the ^18^F-FDG uptake rate (Ki) in the baseline, diluent and allergen lobes (Fig 2) found no significant difference in the Ki between the baseline, diluent and allergen lobes.

**Fig 3:**
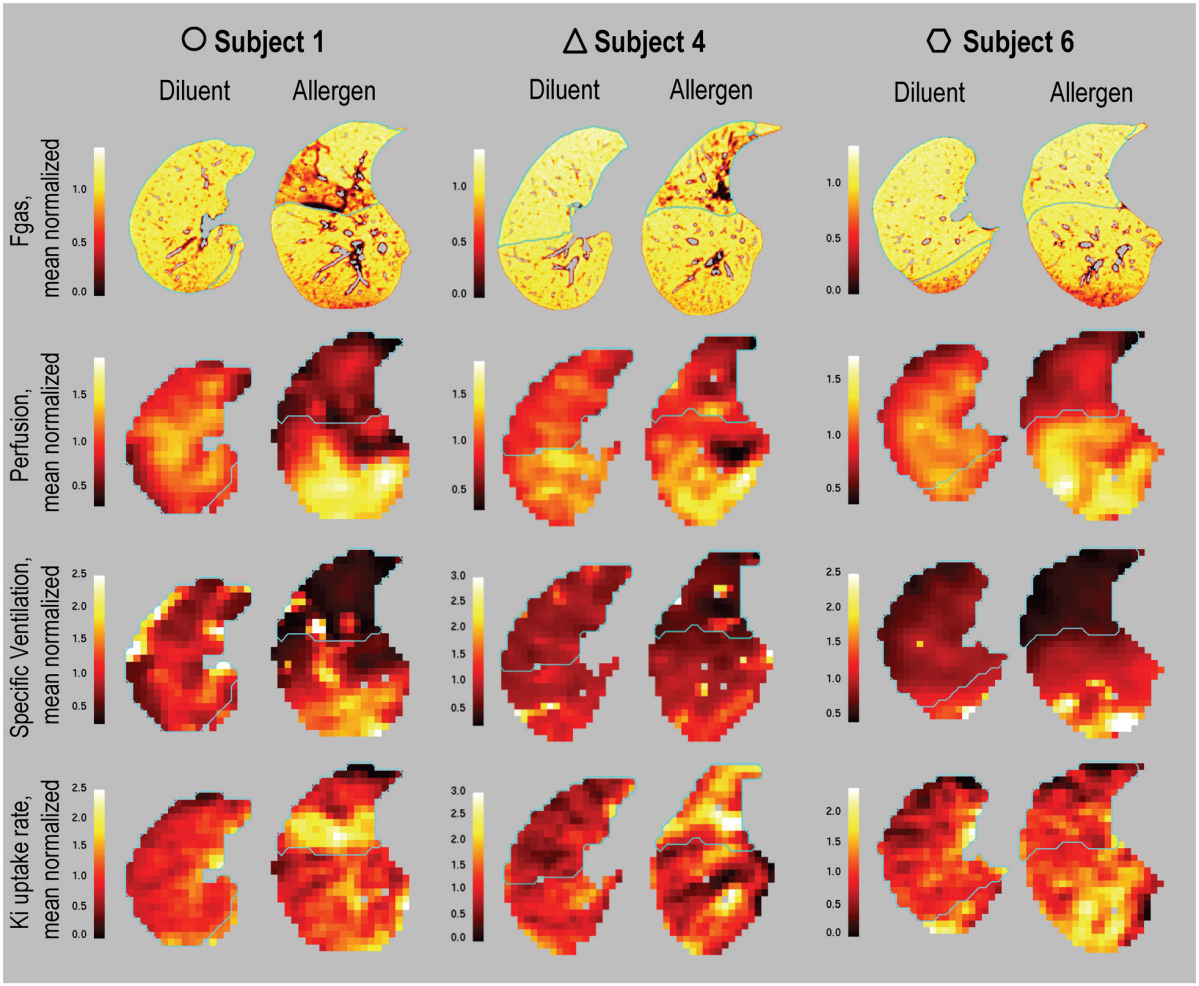
Example images. Representative images of Fgas, Q, sV, and Ki in the diluent and allergen lobes from two subjects who had ground glass opacities present on inspection of HRCT (Subject 1 and 4) and a separate subject with no ground glass opacities (Subject 6). Ground glass opacities are seen as regions with increased red and black colors. In each image, the boundaries of the diluent and allergen lobes are outlined in blue. Note: Color scales differ between subjects. Q, sV and Ki are represented as relative values, relative to the mean of the imaged lung.

Typical regional distributions of Fgas, Q, sV, and Ki in the allergen and diluent lobe in three subjects are shown in Fig 3. Note that the Fgas images show distinct regional alterations in lung aeration in two subjects and no changes in the third subject. Also, perfusion and ventilation images demonstrate substantial differences in the allergen lobe in all three subjects shown, particularly noticeable at the lobe boundary. Finally, the allergen lobe shows higher relative Ki in two subjects but a weaker response is seen in the third subject.

### Cellular outcomes

**Fig 4:**
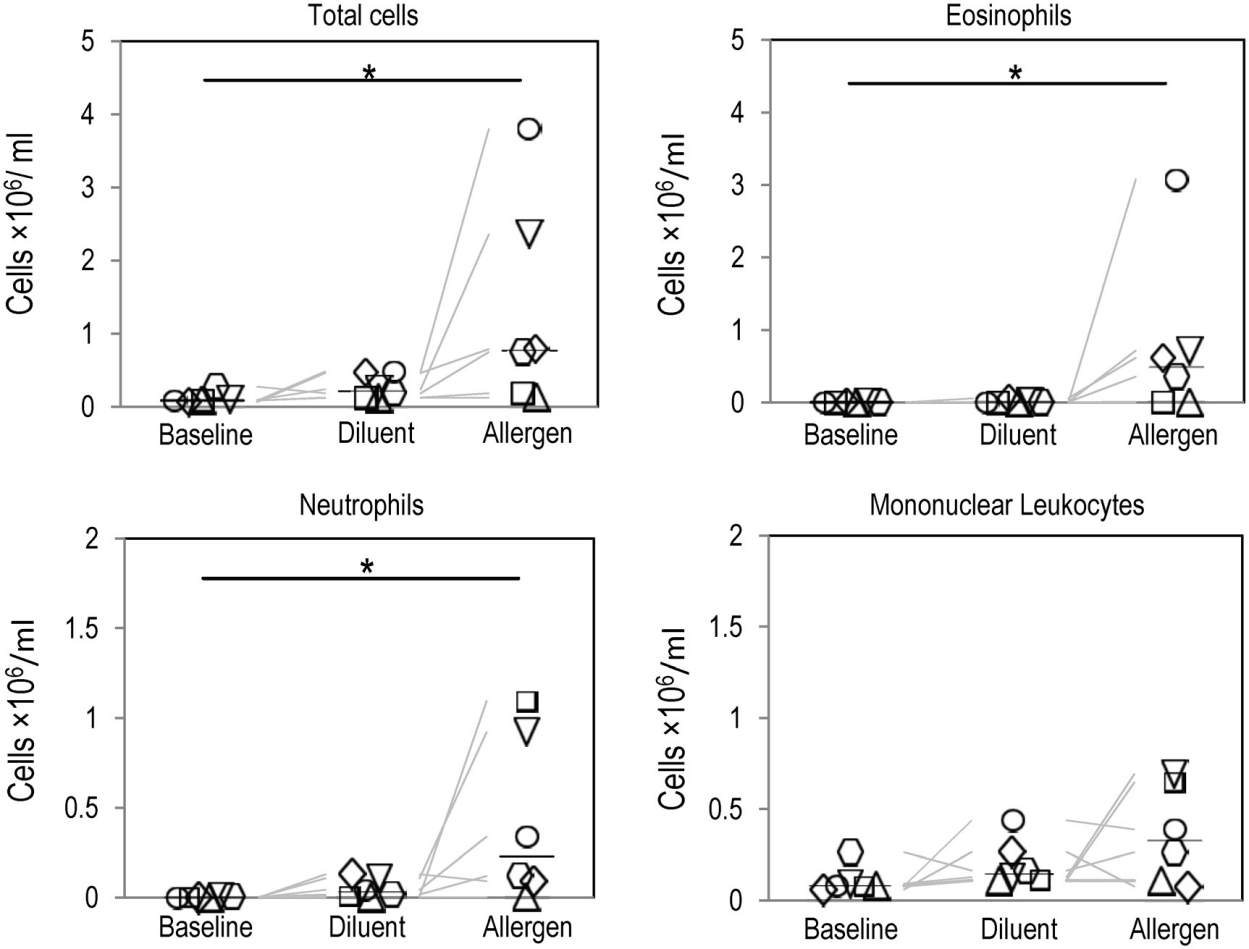
Cellular changes in response to segmental allergen challenge. Comparison of cell counts per ml of BAL fluid in the baseline, diluent and allergen BAL samples. The allergen BAL had significantly greater numbers of total cells, eosinophils, and neutrophils than the baseline BALs. No differences in the mononuclear leukocyte cell counts were found between the baseline, diluent and allergen.

Comparisons of cell counts (per ml of returned bronchoalveolar lavage fluid) between conditions (baseline, diluent and allergen) are included in Fig 4. The allergen BAL had significantly higher total cell counts/ml, eosinophil counts/ml and neutrophil counts/ml compared to the baseline BAL (P<0.05) as determined by cytospins. No differences were found between the baseline, diluent and allergen BAL for mononuclear leukocytes. Furthermore, no significant differences in cell counts between the baseline and diluent or between diluent and allergen lobes were found for any cell type. However, for the allergen lobe total cell counts and eosinophil cell counts were greater than the diluent counts in all subjects.

## Discussion

In this study we found that allergic non-asthmatics can exhibit a regional pulmonary response to segmental allergen challenge even though, clinically, they experience no pulmonary allergic symptoms. Specifically, the allergen lobe was seen to have lower perfusion and ventilation compared to the baseline and diluent lobes, a similar response to that seen in AA subjects following segmental allergen challenge [6]. It follows that AA and ANA subjects are not easily distinguishable based on their regional physiological response to segmental allergen challenge. Given that the cellular allergic inflammation was similar, it could be that the AA phenotype arises from small chronic anatomic differences in the airways between AA and ANA resulting in a lowering of the threshold for the airway tree to undergo “catastrophic” bronchoconstriction in AA [5].

The lower ventilation found in the allergen lobe is likely the result of airway narrowing, particularly in the peripheral airways, which are frequently proposed as being responsible for regional decreases in ventilation [16,17,18]. Peripheral airway narrowing and the associated lower ventilation following segmental challenge could occur as a result of the regional cellular allergic response as indicated by the greater concentrations of total cells, eosinophils and neutrophils (Fig 4). Increased concentrations of these inflammatory cells are associated with increased microvascular leakage and/or cellular exudate production [19,20] and bronchoconstriction, all of which could cause airway narrowing and therefore lower regional ventilation.

In the presence of reduced ventilation, perfusion may also be decreased [4] as low ventilation leads to regional hypoxia, which in turn causes regional vascular constriction likely via hypoxic pulmonary vasoconstriction [21,22]. The reduction of perfusion in regions with reduced ventilation acts to preserve ventilation/perfusion matching and maintain gas exchange, although some degree of mismatch still occurs [23]. Our current findings of lower perfusion and ventilation in the allergen lobe in ANA and our similar findings in a previous study of AA subjects [6], suggests that this effect may be a universal allergic response to segmental allergen challenge in atopic subjects.

The lack of a difference in the degree of lung aeration between the baseline, diluent and allergen lobes suggests that despite the degree of cellular infiltration in the allergen lobe being greater than baseline, this did not consistently lead to regional edema or atelectasis that was visible with HRCT imaging (2/6 subjects had distinct regional reductions in Fgas; Figs 2 and 3). In contrast, lower lung aeration was found previously in the allergen lobe of AA subjects when compared to the diluent and control lobes [6]. The lack of a reduction in lung aeration in the ANA group may be the result of a lower degree of microvascular leakage and/or cellular exudate production in the ANA group following segmental challenge compared to the AA group. However, despite any hypothetical differences in plasma exudation, AA and ANA subjects ultimately experience similar degrees of lower ventilation. Additional, larger studies are warranted to determine whether the degree of microvascular leakage and/or cellular exudation differs between AA and ANA subjects and any role they play in altering lung aeration.

The degree of cellular infiltration following segmental challenge in the ANA subjects reported here was similar to that reported previously in AA subjects. This finding is consistent with the work of Shaver et. al. [24] and Becky Kelly et. al. [25], but this finding is not universal [26,27]. It is possible that absolute cell populations may not be the dominant factor that distinguishes these two clinical phenotypes. Indeed other factors such as the cells capacity to release mediators, their response to local stimulatory signals, or the response of structural cells in the lung to the inflammatory cells may be more relevant. Subsequently, larger studies are required to ascertain the importance of absolute cell populations vs. the functional/metabolic cellular response in the clinical separation of AA and ANA subjects.

### Study limitations

**Fig 5:**
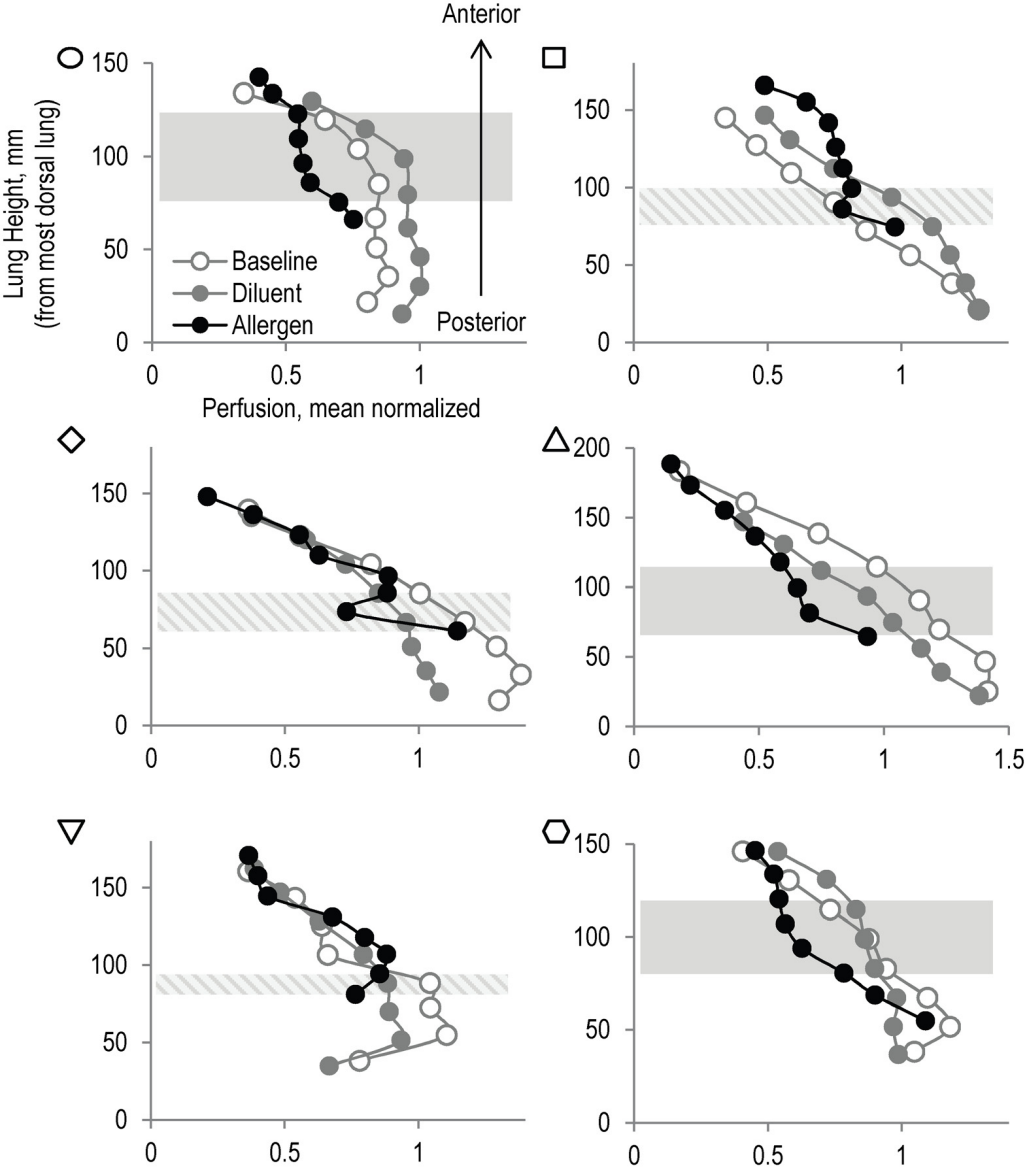
Perfusion relative to gravitational height. Mean normalized perfusion vs. lung height (from the most dorsal point of the lung) in the baseline (grey-open circles), diluent (grey-filled circles) and allergen lobes (black-filled circles) in all subjects (subjects identified by marker in top left of each panel). Each lobe is divided into 8 equal volume regions along the gravitational axis and the mean perfusion and lung height of each region plotted. The allergen lobe can be seen to have lower perfusion across several lung heights (grey highlighted sections), compared to the diluent and allergen. In the remaining 3 subjects the allergen lobe can be seen to deviate to the left of the perfusion height trend for at least one lung height (grey striped highlighted sections). In the most dependent and most non–dependent heights the allergen lobe appears to have no difference in perfusion compared to the baseline and diluent lobes at those equivalent lung heights.

The main limitation of the current study is that the statistical power for differences among parameters is limited as a result of the variability in responses and the small population studied. One additional limitation of our current study is that the baseline, diluent and allergen lobes were not randomized. Such randomization has not previously been included in segmental allergen studies [6,8]. Therefore, systematic inter-lobar differences in perfusion, ventilation and lung aeration may contribute to our findings in two ways. First, because the same volume of allergen/diluent was instilled into lobes of differing size it may have affected the magnitude of the inflammatory responses. Specifically, the diluent lobe has, on average, 1.7 times greater total volume than the allergen lobe, meaning that there is a greater potential for inflammatory cell release due to the greater amount of lung tissue present in the diluent lobe compared to the allergen. Second, the gravitational dependence of perfusion, ventilation and lung aeration is well reported [28,29] and given that the RML is more non-dependent in the supine position than the RUL and LUL, some of the differences in Fgas, perfusion and ventilation in the allergen lobe may be the result of its location. However, the presence of consolidation and ground glass opacities within the RML in a proportion of the ANA subjects (3 of 6; see Fig 2) is almost certainly the result of the instillation of the allergen and therefore it seems unlikely that the reduction in perfusion and ventilation in the allergen lobe can be entirely explained by the specific lobe selected for the segmental challenge. Furthermore, when perfusion in the baseline, diluent and allergen lobes was plotted relative to the lung height (height from most dependent part of the lung) the allergen lobe was seen to have substantially lower perfusion than the control lobes for the same lung height in 3 subjects (Fig 5). In the remaining 3 subjects, perfusion can be seen to deviate from the perfusion vs. height trend for at least one lung height. Therefore, the location of the lobes with respect to gravity is unlikely to account for the systematically lower perfusion in the allergen lobe.

### Physiological Interpretation

Allergic non-asthmatic subjects, following allergen challenge, appear to experience the same physiologic pulmonary disturbances as allergic asthmatic subjects. Therefore, it is not obvious from our findings how ANA subjects are ‘protected’ from lower airway symptoms that result from their allergic disease. One potential explanation is that due to the complex network interactions between airways and alveolar tissue [5], regional lung changes in ANA subjects are able to be compensated by the remaining healthy lung and therefore pulmonary symptoms may not manifest. Factors such as airway remodelling in AA subjects [30,31] may mean the normal compensatory mechanisms do not work and therefore AA subjects experience pulmonary symptoms such as shortness of breath.

### Conclusion

Despite having no clinical expression of airways disease, ANA subjects respond to segmental allergen challenge with regional changes in perfusion, ventilation and cell counts that are similar to those that occur in AA subjects. It follows that the mechanisms which protect ANA subjects from airways disease must differ from the parameters investigated herein. Additional studies will be necessary to clarify if there is a quantitative difference in the magnitude of the pulmonary response between asthmatic and non-asthmatic patients with allergies that may explain the differences in the clinical manifestation of their allergic disease.
